# *Theobroma cacao* seed extracts attenuate dyslipidemia and oxidative stress in L-NAME induced hypertension in Wistar rats

**DOI:** 10.7150/ijms.123888

**Published:** 2025-10-27

**Authors:** Eyuwa I. Agwupuye, Abdulhakeem R. Agboola, Yu-Cheng Kuo, Ako H. Itam, Lawrence U. Ezeayinka, Item J. Atangwho, Bolaji M. Ayeyemi, Adeleye A. Edema, Bashir Lawal, Modhi O. Alotaibi, Najlaa Hamed Almohmadi, Gaber El-Saber Batiha, Nasser M. El-Sabbagh, Hsu-Shan Huang

**Affiliations:** 1 Department of Biochemistry, College of Medicine, University of Calabar, Calabar, Cross River State, Nigeria.; 2 Department of Pharmacology, School of Medicine, College of Medicine, Taipei Medical University, Taipei, 11031, Taiwan.; 3 School of Post-Baccalaureate Chinese Medicine, College of Chinese Medicine, China Medical University, Taichung, 40604, Taiwan.; 4 Department of Internal Medicine, College of Medicine, University of Calabar, Calabar, Cross River State, Nigeria.; 5 Department of Biochemistry, Faculty of Biological Sciences, University of Nigeria, Nsukka, Nigeria.; 6 North Carolina Agricultural and Technical State University, USA.; 7 Department of Chemistry and Biochemistry, The University of Tulsa, USA.; 8 UPMC Hillman Cancer Centre, USA.; 9 Department of Biology, College of Science, Princess Nourah bint Abdulrahman University, P.O. Box 84428, Riyadh 11671, Saudi Arabia.; 10 Environmental and biomaterial Unit, Natural and Health Sciences Research Center, Princess Nourah bint Abdulrahman University, Riyadh, Saudi Arabia.; 11 Clinical Nutrition Department College of Applied Medical Sciences Umm Al-Qura University, Makkah 24381 Saudi Arabia.; 12 Department of Pharmacology and Therapeutics, Faculty of Veterinary Medicine, Damanhour University, Damanhour 22511, AlBeheira, Egypt.; 13 Department of Veterinary Pharmacology, Faculty of Veterinary Medicine, Alexandria University, Alexandria, Egypt.; 14 School of Pharmacy, National Defense Medical University, Taipei, 11490, Taiwan.; 15 PhD Program for Cancer Molecular Biology and Drug Discovery, College of Medical Science and Technology, Taipei Medical University, Taipei, 11031, Taiwan.; 16 Graduate Institute of Cancer Biology and Drug Discovery, College of Medical Science and Technology, Taipei Medical University, Taipei, 11031, Taiwan.; 17 Ph.D. Program in Biotechnology Research and Development, College of Pharmacy, Taipei Medical University, Taipei, 11031, Taiwan.

**Keywords:** Oxidative stress, antioxidants, L-name, DPPH, Nitric oxide, lipid profile

## Abstract

This research evaluated the antioxidant activities (*in vitro* and *in vivo)* and serum lipid profile of the chloroform (CETC) and ethanol (EETC) extracts of *Theobroma cacao* seeds in nitro-L-arginine methyl esters (L-NAME)-induced hypertension in Wister rats. Parameters investigated include DPPH, nitric oxide scavenging ability, CAT, GPX, SOD and serum MDA concentration. Serum lipid profiles such as total cholesterol, HDL, LDL, VLDL and TG were also assayed. The rats were treated with 100mg/kg, 200mg/kg and 300mg/kg b. w of ethanol and chloroform extracts of *T*. *cacao* and a standard drug concurrently with L-NAME. The results showed a dose-dependent increase in the antioxidant activities of EETC and CETC across all the invitro parameters with the ethanol extracts consistently outperforming the chloroform extracts. Total cholesterol, low-density lipoproteins, very low-density lipoprotein, TGs, and serum MDA showed significant (p<0.05) decreases in comparison with the control. On the other hand, there were significant (p<0.05) increases in high-density lipoproteins, GPx, SOD, CAT, and serum Nitric Oxide in the treated rats relative to the untreated rats. The observed antihypertensive properties of the extracts support that *T. cacao* may be useful in the management of cardiovascular health. The active constituents of these extracts could protect against hypertension.

## Introduction

Hypertension, a persistent elevation of blood pressure, is a major risk factor for cardiovascular diseases (CVDs), which account for about 31% of global mortality 1. Affecting an estimated 1.4 billion people worldwide, it remains a “silent killer,” with most individuals unaware of their condition and fewer than one in five achieving adequate control 1,2. In 2017, hypertension contributed to 10.4 million deaths, largely through its role in coronary artery disease, stroke, and chronic kidney disease 3,4. Dyslipidemia, defined by low high-density lipoprotein (HDL) and elevated triglycerides, total cholesterol, and low-density lipoprotein cholesterol (LDL-C), further heightens the risk of CVDs, particularly coronary artery disease and atherosclerosis 5,6.

Oxidative stress and inflammation underlie metabolic disorders including dyslipidemia, hypertension, and glucose intolerance 7. Excess reactive oxygen species (ROS) promote cellular damage and inflammatory responses 8, while persistent low-grade inflammation accelerates atherosclerosis through fibrofatty plaque formation that develops early and remains silent until symptomatic 9. Given the interplay of oxidative stress, inflammation, dyslipidemia, hypertension, and cardiovascular disease, antioxidants are considered pivotal in managing these disorders. By neutralizing free radicals, antioxidants limit oxidative damage and slow disease progression, even at low concentrations 10. The rising burden of chronic conditions such as diabetes and inflammation-related disorders has intensified interest in natural sources of antioxidants. Medicinal plants, rich in bioactive phytochemicals, are particularly attractive due to their multitarget pharmacological effects and minimal side effects 11

For centuries, cocoa-rich chocolate has been valued not only for its taste but also for its health benefits. The Incas revered cocoa as the “drink of the gods,” inspiring the scientific name *Theobroma cacao*—from the Greek *theo* (god) and *broma* (drink) 12. Its benefits are linked to bioactive compounds such as flavonoids (epicatechin, catechin, procyanidins), theobromine, and caffeine. Flavonoids provide antioxidant and anti-inflammatory effects, enhance endothelial nitric oxide-mediated vasodilation, and support lipid metabolism, while theobromine offers mild vasodilatory and diuretic actions that aid blood pressure regulation. Together, these mechanisms underpin cocoa's cardiovascular and metabolic protective effects. Historically, cocoa was also used to treat conditions including angina, anemia, mental fatigue, fever, gout, kidney stones, and digestive disorders 12. This study aims to offer novel insights into the medicinal properties of ethanol and chloroform extracts of Theobroma cacao to validate its acclaimed potency in the management of hypertension and its associated complications and related diseases.

## Materials and Methods

### Chemicals and reagents

All chemicals and reagents used in this research were of analytical grade. Standard commercial test kits of Randox (Randox Laboratories Limited, 55 Diamond Road, Crumlin, Co. Antrim, United Kingdom, BT29 4QY), TECO (1268 Lakeview Avenue, Anaheim CA 92807 USA), QCA (P. O. Box 20, 43870 Amposta, Spain) and Spinreat (SPINREACT, S. A. Ctra.Santa Coloma, 7 E-17176 SANT ESTEVE DE BAS GI, Spain) were also used for the study.

### Collection and extraction of plant material

Cocoa beans were collected from Bendeghe Afi village and were further processed in Ukwu Investment Limited, Ikom, Cross River State, Nigeria. The identification and certification of the seeds were carried out at the Department of Plant and Ecological Studies, University of Calabar. Subsequently the Dried cocoa beans were sun dried for about 14 days. The seeds were de-coated, ground and sieved into fine powder (cocoa powder). The ethanol and chloroform extracts of *Theobroma cacao* were prepared by macerating 1000 g of cocoa powder in 3500 ml of each solvent for 48 hours. The mixtures were then filtered through Whatman No. 1 filter paper, and the filtrates concentrated under reduced pressure. The extraction yielded 7.5% (ethanol) and 5.7% (chloroform), and the extracts were stored in sterile bottles until use.

### Evaluation of *in vitro* antioxidant activities

#### Nitric oxide radical scavenging assay

The *in vitro* nitric oxide (NO) radical scavenging activity was evaluated using the procedure described by Tonisi 13. The reaction mixture consisted of 2.0 mL of sodium nitroprusside, 0.5 mL of phosphate-buffered saline (PBS), and 0.5 mL of the seed sample (50 mg). The mixture was incubated at 25 °C for 30 minutes to initiate NO generation. Subsequently, 0.5 mL of Griess reagent was added, and the mixture was further incubated for another 30 minutes. A freshly prepared control (without the sample) was included, and the absorbance of both the sample and the control was measured at 546 nm. The Griess reagent served as the blank. The percentage inhibition of nitric oxide production was calculated to assess the NO scavenging activity of the sample.

% Inhibition of NO = 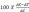


Where: AC = Absorbance of Control; AT = Absorbance of Test.

#### Diphenyl-1-picrylhydrazyl (DPPH) radical scavenging activity

The antioxidant activity of the sample was assessed using the 2,2-diphenyl-1-picrylhydrazyl (DPPH) radical scavenging assay, following the method described by Blois et al. 14. Sample solutions at various concentrations were prepared in ethanol. Each sample solution was mixed in a 1:1 ratio with a 0.2 mM DPPH solution prepared in ethanol. The reaction mixtures were incubated in the dark at room temperature for 30 minutes. After incubation, the absorbance of each mixture was measured at 517 nm. A blank (ethanol and DPPH solution without the sample) was used for comparison. The DPPH radical scavenging activity was calculated using the following formula:

Percentage inhibition (%) = 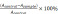


where A control is the absorbance of the DPPH solution without sample, and A sample is the absorbance in the presence of the test sample. Measurements were done in triplicates.

#### Hydrogen peroxide (H₂O₂) scavenging activity

The hydrogen peroxide (H₂O₂) scavenging capacity of the extracts were determined using the method described by Ruch et al. 15. A 43 mM H₂O₂ solution was prepared in 0.1 M phosphate buffer (pH 7.4). Various concentrations of the seed extracts were mixed with the phosphate buffer and subsequently added to the H₂O₂ solution. The reaction mixtures were incubated for 10 minutes at room temperature, after which the absorbance was measured at 230 nm against a blank containing phosphate buffer without H₂O₂. The percentage scavenging of hydrogen peroxide was calculated using the following formula:



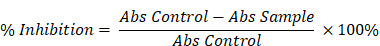



### Heat induced hemolysis

Heat-induced hemolysis was assessed following the methodology described by Juvekar et al. 16. The reaction mixture consisted of 2 mL total volume, comprising 1 mL of seed extract and 1 mL of a 10% red blood cell (RBC) suspension obtained from Albino Wistar rats. For the assay, normal saline served as the control, while diclofenac was used as the reference standard. All samples were incubated at 56 °C for 30 minutes in a water bath. After incubation, the tubes were cooled under running tap water and centrifuged at 2500 rpm for 5 minutes. The absorbance of the supernatant was measured at 560 nm. All experiments were performed in triplicate to ensure reliability of the results.



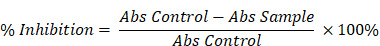



### Proteinase inhibitory assay

The proteinase inhibitory activity of the seed extract was assessed using the method described by Oyedepo and Femurewa 17. The reaction mixture (2 mL total volume) contained 0.06 mg of trypsin and 1 mL of Tris-HCl buffer, which was incubated with 1 mL of the seed extract at 37 °C for 5 minutes. Following incubation, 0.8% (w/v) casein was added as the substrate, and the mixture was further incubated for 20 minutes at the same temperature. The reaction was terminated by adding 2 mL of 70% perchloric acid. After centrifugation, the absorbance of the resulting supernatant was measured at 210 nm. The degree of proteinase inhibition was determined by comparing the absorbance values of the test samples with that of the control (without extract).

### Inhibition of protein denaturation

The inhibition of protein denaturation was evaluated using the method described by Mizushima and Kobayashi 18, with slight modifications. Briefly, various concentrations of the extract or standard drug (diclofenac) were mixed with 1% (w/v) bovine serum albumin (BSA) prepared in distilled water. The reaction mixtures were incubated at 55 °C for 30 minutes to induce protein denaturation, followed by cooling to room temperature. The absorbance of each sample was measured at 660 nm using a UV-Visible spectrophotometer.

### Animals used in the experiment

A total of 81 adult male albino rats weighing 200 and 250g were used in the study. All the animals used were obtained from the Animal House of the Department of Zoology, University of Nigeria, Nsukka. The animals were housed in an ambient temperature, relative humidity and light and dark circle (12 h light/dark). The rats were fed with standard rat pellets and water *ad libitum*. The guide for the care and uses of laboratory animal's procedure were followed in this study (National Institutes of Health (NIH) publication (1985) for the care and use of laboratory animals). Ethical approval was obtained from the faculty animal research ethics committee of the Faculty of Basic Medical Sciences (FAREC-FBMS) University of Calabar with the approval number: 022-TBCM-0521.

### Experimental design

A total of 81 male Wistar rats weighing 200-350 g were used for the study. They were acclimatized for 14 days with free access to water and feed. After acclimatization, they were allocated based on weight similarity into nine study groups of nine rats each. The baseline blood sample levels were determined before administration of the extracts. The treatment lasted for 21 days after which the blood samples of the rats were taken on zero, 14 and 21 days. The route of administration was via oral route with the aid of oral gastric tube. The groups and doses administered are summarized below.

Group 1: Feed + water (normal control). Group 2: L-NAME only (hypertensive control). Group 3: L-NAME + 100 mg/kg of ethanol extract. Group 4: L-NAME + 200 mg/kg of ethanol extract. Group 5: L-NAME + 300 mg/kg of ethanol extract. Group 6: L-NAME + 100 mg/kg of chloroform extract. Group 7: L-NAME + 200 mg/kg of chloroform extract. Group 8: L-NAME + 300 mg/kg of chloroform extract. Group 9: L-NAME + standard drug (nifedipine).

### Measurement of blood pressure

The baseline blood pressure was measured and recorded; subsequent measurements were done before blood collection. This was done by tail-cuff method using non-invasive Ugo Basile, Series 58500 blood pressure recorder and invasive method using one-unit channel recorder calibrated with a sphygmomanometer. Averages of three readings were taken for each rat and the temperature of the rat was monitored throughout the measurement period. Mean arterial blood pressure was calculated according to the following equation: DP + 1/3 (SP-DP) where SP and DP are systolic and diastolic pressures respectively 19.

### Collection of blood sample

Baseline analyses of the biochemical parameters of the rats were carried out before the administration of the extracts of Theobroma Cacao. In each of the blood collections, three animals were sacrificed, and blood samples were obtained from abdominal aorta using syringe.

### Induction of hypertension and administration of the extracts

L-NAME (Nitro-L-arginine methyl ester) dissolved in distilled water was administered orally at a dose of 40 mg/kg body weight using an oral feeding gavage. Administration was done concurrently with the test extracts. Ethanol and chloroform extracts of *Theobroma cacao* (125 mg) were each dissolved in 2.5 mL of distilled water. The extracts were administered orally to the experimental animals at doses of 100, 200, and 300 mg/kg body weight once daily for 21 consecutive days. Blood samples were collected via the abdominal aorta using syringe on days 14 and 21. The sera were separated and stored for subsequent biochemical analysis. The experiment was terminated after the final blood collection on day 21.

### Serum lipid profile

#### Determination of total cholesterol

The cholesterol assay was carried out using QCA (Quimica Clinica Aplicada S.A., a Spanish manufacturing company) commercial kit according to the method described by Fiedewald *et al.* 20. The procedure for the assay was according to the manufacturer's instruction contained in the kit insert.

#### Determination of high-density lipoprotein cholesterol (HDL-c)

The high-density lipoprotein cholesterol (HDL-c) assay was carried out using a QCA (Quimica Clinica Aplicada S.A., a Spanish manufacturing company) commercial kits according to the method described by Albers et al. 21. The procedure for the assay was according to the manufacturer's instruction contained in the kit insert.

#### Determination of serum triacylglycerol concentration

Triacylglycerol assay was determined using a Randox commercial kit, The procedure for the assay was according to the manufacturer's instruction contained in the kit insert.

#### Determination of serum low-density lipoprotein cholesterol (LDL-c) concentration

Low-density lipoprotein was calculated using the Friedewald equation namely LDL = TC - (TG/5 + HDL-c). Where TC = Total cholesterol, TG = Triacylglycerol and HDL-c = High density lipoprotein cholesterol 20.

#### Determination of very low-density lipoprotein cholesterol (VLDL-c) concentration

Very low-density lipoprotein cholesterol concentration was calculated using the equation of Friedewald: Triacylglycerol / 2.238 = 'very low-density lipoprotein 20.

### Serum antioxidant indices

#### Determination of glutathione peroxidase activity

Glutathione peroxidase (GPx) activity was determined according to the method of Bergmeyer 22. The principle is based on the oxidation of pyrogallol to purpurogallin by the peroxidase in the sample, resulting in formation of a deep brown coloration which is read at 430 nm. Briefly, 0.2 mL of **20** mM pyrogallol (0.2552g of pyrogallol was dissolved in 100 mL of distilled water). 2.5 mL of phosphate buffer (pH), 2.5 mL of H_2_O_2_, and 1.5 mL of pyrogallol were added in a test tube. The reaction mixture was allowed to stand for 30 minutes at room temperature, and the absorbance measured at 430 nm. The activity of GPx per unit protein content of the sample, was then calculated thus:

GPx activity (unit) = AT x vt x Df / E x Vs x Y

Where: AT = Absorbance of the test sample; Vt = Total volume of reaction mixture; Df = Dilution factor = 1; E = Molar extinction co-efficient (12M/cm); Vs = Volume of sample used; Y = mg of protein.

### Determination of superoxide dismutase activity

Superoxide dismutase (SOD) activity was determined by the method of Misra and Fridovich 23. Adrenaline undergoes rapid auto-oxidation in the presence of superoxide anions to form adrenochrome, whose concentration can be determined at 420nm with the aid of a spectrophotometer. Superoxide dismutase inhibits this auto-oxidation of adrenaline by catalysing the breakdown of superoxide anions. Therefore, the degree of inhibition reflects the activity of SOD in the sample, which is determined at 420nm.To 0.2 mL of serum was added 2.5 ml of carbonate buffer (0.05M, pH 10.20 and 0.3 mL of freshly prepared 0.3 mM adrenaline solution in HCl (10.98 mg of adrenaline in 10 mL of 0.005M HCl) and absorbance read at 420 nm. The absorbance of a reference/blank sample comprised of 0.2ml of distilled water plus 2.5 mL of carbonate buffer and 0.3 mL of adrenaline is also measured and used in estimation of enzyme activity. The results, being mean of three determinations, were expressed in units/mg protein thus:

% Inhibition = (At - A ref** )** x 100 / At

The SOD activity can then be calculated:

Unit/mg protein = % inhibition / 50 x Y

Where: At = Absorbance of test; A ref = Absorbance of reference sample; Y = mg protein in the volume of the sample used.

### Determination of catalase (CAT) activity

Catalase activity was determined using a Randox commercial kit, the procedure for the assay was according to the manufacturer's instruction contained in the kit insert.

### Determination of malondialdehyde (MDA) concentration

The lipid peroxidation product, MDA was assayed by the method described by Wallin *et al*. 24 The principle is based on the formation of a red or pink complex which absorbs maximally at 532 nm when MDA radicals in the sample react with thiobarbituric acid (TBARS) in an acidic medium.

Briefly, 0.01 mL of sample was added to 0.5 mL of 25% TCA and 0.5 mL of 1% thiobarbituric acid and mixed thoroughly. A reagent blank solution was prepared with the same volume of TCA and TBA, but with 0.1 mL of distilled water in place of sample. These reaction media were heated in a water bath at 95 ^°^C for 40 minutes, with turbidity removed by centrifugation. The sample mixture on cooling had its absorbance read against that of the reagent blank at 532 nm. The results were expressed as mg/protein using the molar extinction coefficient of 1.56 x 10^5^/M cm. The concentration of MDA was determined using the formula:

MDA (unit/mg protein) = A x V x 1000 / M × v × y

Where: A = Absorbance; V = Total Volume of reaction mixture; M = Molar extinction co-efficient; v = Volume of the sample; y = mg protein.

### Determination of nitric oxide concentration

Nitric oxide was assayed by the method of Marcocci *et al.* 25. In the procedure, 1 mL of the reaction mixture contained 500 µL of serum in dimethylsulfoxide (DMSO) saline mixed with 5mM sodium nitroprusside prepared in 10 mM potassium phosphate buffer (pH 7.4), then incubated at 25 ^°^C for 150 minutes. At the end of incubation, the samples were allowed to react with 1 mL of Greiss reagent containing an equal volume of solution A (1 % sulfanilamide and 5 % H_3_PO_4_) and B (0.1 % naphthyl ethylenediamine dihydrochloride). The absorbance of the chromophore formed during the diazotization of nitrite with sulfanilamide and subsequent coupling with naphthyl ethylenediamine was read at 540 nm.

A_0_ - A_1_/A_0_x100

Where: A_0_ = Absorbance of control; A_1_ = Absorbance of Sample

### Statistical analysis

All data were expressed as mean ± standard error of the mean (SEM). Statistical comparisons between groups were performed using one-way analysis of variance (ANOVA), followed by Tukey's post hoc test. A p-value of less than 0.05 was considered statistically significant.

## Results

### *In vitro* antioxidant activities of the extracts

#### Nitric oxide activity

**Figure 1:**
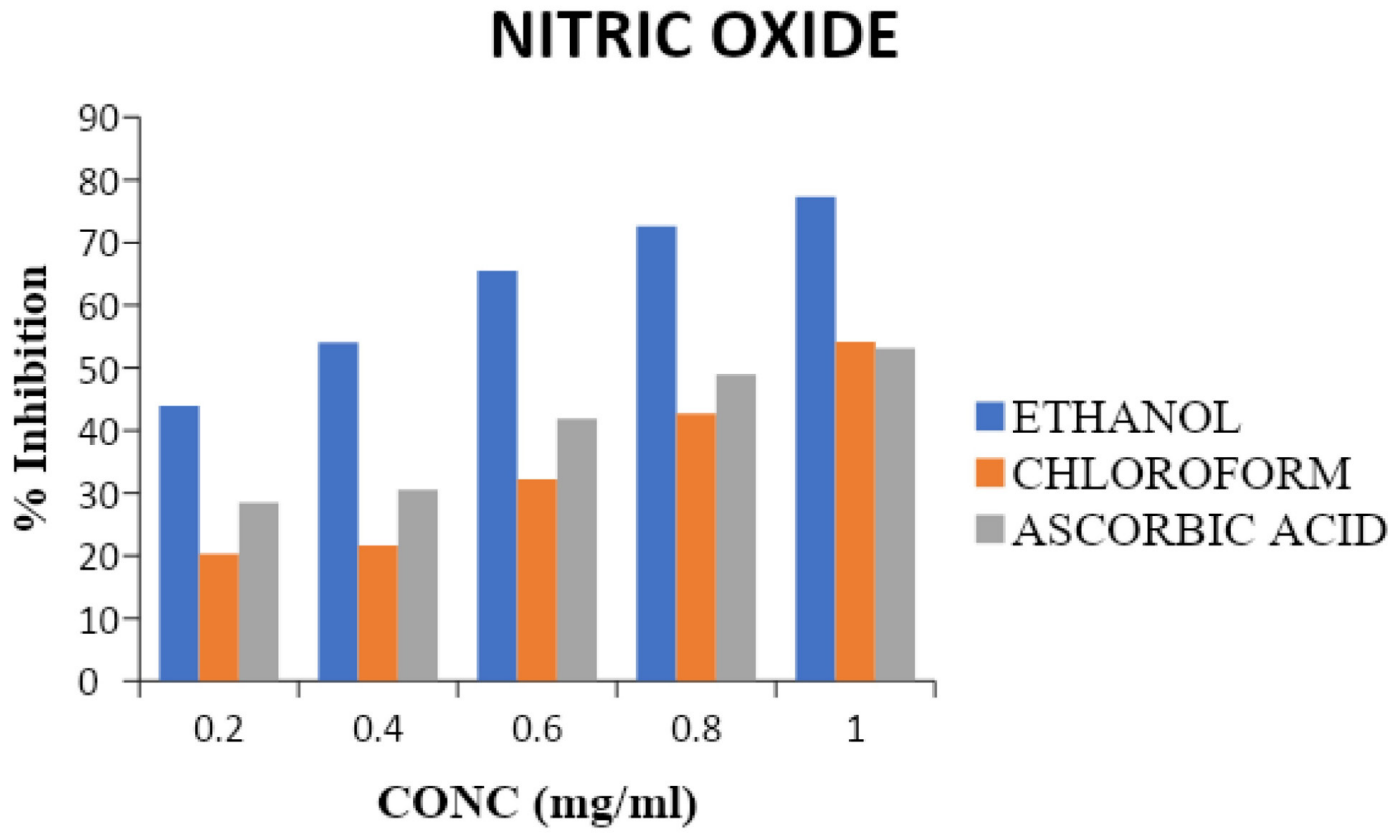
Percentage scavenging activity of chloroform and ethanol extracts of *T. cacao* on nitric oxide (Average of triplicate experiments). Mean ± SD, n = 3. Statistical significance was assessed using one-way ANOVA followed by Tukey's post hoc test; p-values < 0.05 were considered significant. Statistical significance was assessed using one-way ANOVA followed by Tukey's post hoc test; p-values < 0.05 were considered significant.

The incubation of sodium nitroprusside solutions in phosphate saline at 25 ºC for 150 min resulted in linear time-dependent nitrite production, which was reduced by both the ethanol and chloroform extracts of *T. cacao.* The ethanol extract exerted a greater inhibition value (77.3%) compared to the chloroform extract and ascorbic acid. The maximum nitric oxide scavenging of chloroform extract and ascorbic acid was 54.1 % and 53.1 %, respectively, as shown on Figure 1.

#### DPPH, ABTS, and hydrogen peroxide (H₂O₂) scavenging activities

**Figure 2:**
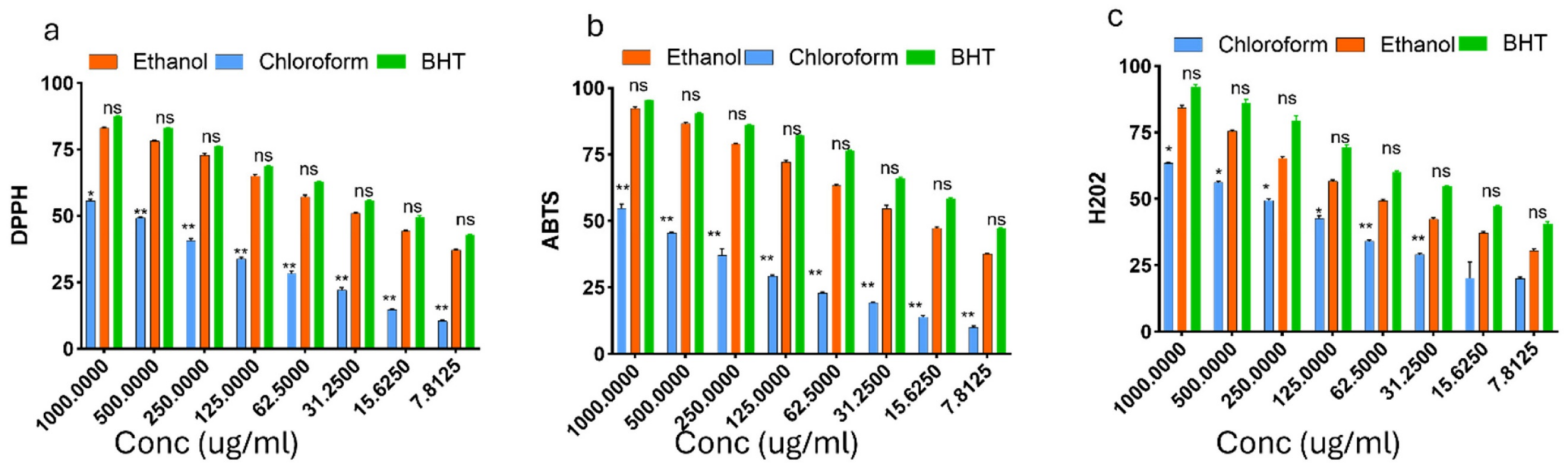
Percentage scavenging activity of ethanol extract of *Theobroma cacao* (EETC), chloroform extract of *Theobroma cacao* (CETC), and butylated hydroxytoluene (BHT, standard antioxidant) against 2,2-diphenyl-1-picrylhydrazyl (DPPH), 2,2′-azino-bis(3-ethylbenzothiazoline-6-sulfonic acid) (ABTS), and hydrogen peroxide (H₂O₂) radicals. Mean ± SD, n = 3 Data are expressed as percentage inhibition (mean ± SEM, n = 3). Statistical significance was assessed using one-way ANOVA followed by Tukey's post hoc test; p-values < 0.05 were considered significant.

Antioxidant activity of chloroform and ethanol extracts of *T. cacao* compared with BHT across different concentrations. (a) DPPH radical scavenging activity, (b) ABTS radical cation decolorization assay, and (c) Hydrogen peroxide (H₂O₂) scavenging activity are presented in Figure 2. Extracts were tested at concentrations ranging from 1000 to 7.8125 μg/mL. Ethanol extract consistently exhibited stronger antioxidant effects than the chloroform extract in all assays. BHT served as the positive control.

### Anti-inflammatory activities

**Figure 3:**
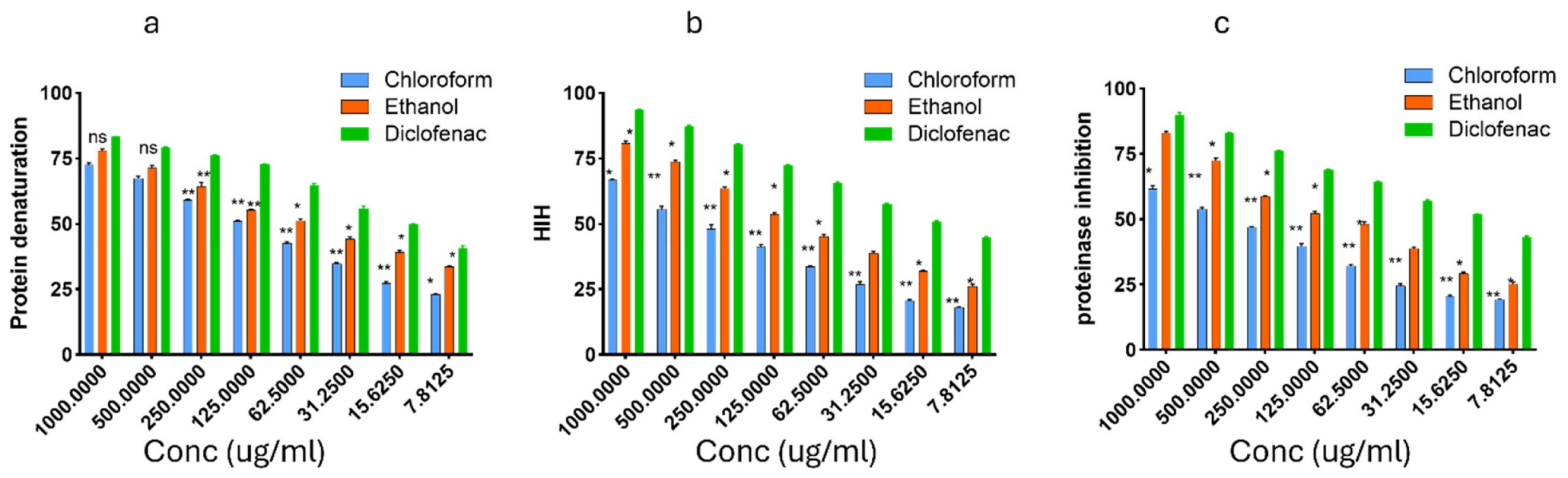
The activities of chloroform and ethanol extracts of *T. cacao* on Inhibition of protein denaturation, Heat-induced hemolysis (HIH), and Proteinase inhibitory activity activities. Data are presented as mean ± SEM (n = 3). Statistical analysis was performed using one-way ANOVA and Tukey's multiple comparison test; significance was defined as p < 0.05.

*In vitro* anti-inflammatory activities of chloroform and ethanol extracts of *T. cacao* compared with diclofenac sodium. (a) Inhibition of protein denaturation, (b) Heat-induced hemolysis (HIH), and (c) Proteinase inhibitory activity are presented in Figure 3 below. Extracts were tested at concentrations between 1000 and 7.8125 μg/mL. The ethanol extract exhibited greater inhibition than the chloroform extract across all assays, with effects generally approaching those of diclofenac.

### *In vivo* antioxidant potential of *T. cacao* extracts

#### *Theobroma cacao* extracts reduced malondialdehyde levels in L-NAME induced hypertension

The effects of *T. cacao* extracts on MDA levels in hypertensive rats are presented in Table 1. The values obtained shows significant (p < 0.05) increase in malondialdehyde levels in rats with untreated hypertensive condition (Group 2) compared with the Group 1 (control) and baseline. However, concurrent treatments of L-NAME with the ethanol extract (groups 3, 4, and 5), chloroform extract (Groups 6, 7, and 8) and Nifedipine (Group 9) showed significant (p <0.05) decreases in malondialdehyde levels in rats relative to that of Group 2 (column) and their baselines (across the rows). Comparing between 14th and 21st days, Group 2 showed a significant (p < 0.05) increase while Groups 3, 4, 5, 8 and 9 showed significant (p < 0.05) decreases as well as non-significant (p > 0.05) differences were observed in groups 6 and 7.

#### *Theobroma cacao* extracts restored GPx levels in L-NAME induced hypertension

The effects of *T. cacao* extracts on GPx levels in hypertensive rats are presented in Table 2. Values obtained showed that there was a decrease in glutathione peroxidase activities in rats with hypertensive conditions (group 2) but there was no significant difference (P>0.05) in comparison to that of the control (group 1). However, treatments with ethanol extract (groups 3, 4, 5), chloroform extract (groups 6, 7, 8) and Nifedipine (group 9) showed a significant increase (P≤0.05) in glutathione peroxidase levels in rats when compared to group 2 (hypertensive untreated). In comparison between baseline, day 14 and day 21, group 2 showed no significant difference (P>0.05) between baseline and day 14 but there was a significant decrease (P<0.05) between baseline and day 21 of administrations, group 3, 8 and 9 showed a significant increase (P>0.05) between day 14 and day 21 of treatments, group 4, 5 and 7 showed no significant difference (P>0.05) between baseline, day 14 and day 21 but there was a significant increase (P≤0.05) between baseline and day 21 of treatments.

#### *Theobroma cacao* extracts restored superoxide dismutase activities in L-NAME induced hypertension

The effects of *T. cacao* extracts on SOD levels in hypertensive rats are presented in Table 3. Values obtained showed that there was a decrease in superoxide dismutase activities in rats with untreated hypertensive conditions (group 2) but there was no significant difference (P>0.05) relative to the control (group 1). However, treatment with ethanol extracts (groups 3, 4, and 5), chloroform extract (groups 6, 7 and 8) and nifedipine (group 9) showed that groups 5, 7 and 8 gave a significant increase (P≤0.05) in superoxide dismutase activities while groups 3, 4, 6 and 9 showed increased activities but there was no significant difference (P>0.05) in comparison to that of group 2 (hypertensive untreated). In comparison between baseline, day 14 and day 21, group 2 showed no significant difference (P≤0.05) between baseline and day 14, between day 14 and day 21 but there was a significant decrease (P<0.05) between baseline and day 21 of administrations, group 3, 4, 5, 6, 7, 8, and 9 showed no significant difference (P>0.05) between baseline, day 14 and day 21 of treatments.

#### *Theobroma cacao* extracts restored catalase activities in L-NAME induced hypertension

The effects of *T. cacao* extracts on catalase levels in hypertensive rats are presented in Table 4. The values obtained showed that there was a significant decrease (P≤0.05) in catalase activities in rats with untreated hypertensive conditions (group 2) in comparison to that of the control (group 1). Treatments with ethanol extract (groups 3, 4 and 5 with 100mg/kg b.w, 200mg/kg b.w and 300mg/kg b.w respectively), chloroform extract (groups 6, 7 and 8 treated with 100mg/kg b.w, 200mg/kg b.w and 300mg/kg b.w respectively) showed a significant increase (P≤0.05) in catalase activities, particularly, group 5 between baseline, day 14 and day 21 of treatments. In comparison between baseline, day 14 and day 21, group 2 showed a significant decrease (P≤0.05) between baseline, day 14 and day 21 but there was no significant difference (P>0.05) between day 14 and day 21 in catalase activities, groups 3, 4, 5, 6, 7, 8 and 9 showed no significant difference (P>0.05) in catalase activities between baseline, day14 and day21.

#### *Theobroma cacao* extracts restored nitric oxide levels L-NAME induced hypertension

The effects of *T. cacao* extracts on catalase levels in hypertensive rats are presented in Table 5. The values obtained shows a significant (p < 0.05) decrease in nitric oxide levels in rats with untreated hypertensive condition (Group 2) compared with the control and baseline. However, concurrent administrations of L-NAME with the ethanol extract (Groups 3, 4 and 5), chloroform extract (Groups 6, 7 and 8) and nifedipine (Group 9) showed a significant (p < 0.05) increases in Nitric oxide levels in rats compared with Group 2 (hypertensive untreated) and their baselines. All the treated groups increased significantly (p <0.05) between 14th and 21st days of administrations.

### Lipid profile

#### *Theobroma cacao* extracts lowered cholesterol concentrations L-NAME induced hypertension

The effects of *T. cacao* extracts on cholesterol levels in hypertensive rats are presented in Table 6. The values obtained results shows induction of hypertension (group 2) gave a significant increase across the row relative to baseline, between day 14^th^ and day 21^st^ and column relative to the control (group 1). However, treatment with the three dose levels of both ethanol (group 3, 4 and 5) and chloroform extracts (group 6, 7 and 8) and nifedipine (group 9) concurrent with L-NAME showed modulation in cholesterol levels after 14 and 21 days. Total cholesterol levels in all the treated groups were significantly (p<0.05) reduced compared with the hypertensive untreated (group 2) and across the rows relative to their baseline but there were no significant (p>0.05) differences between 14^th^ and 21^st^ days except group 9 that showed a significant decrease between day 14^th^ and 21^st^_._

#### *Theobroma cacao* extracts restored HDL levels in L-NAME induced hypertension

Induction of hypertension with L-NAME (Group 2) decreased significantly (P<0.05) when compared with Group 1 and baseline. However, concurrent treatments of L-NAME with ethanol extract, chloroform extract, and nifedipine induced significant (p <0.05) increases in HDL levels at 14th and 21 days relative to Group 2 (hypertensive untreated). Groups 3, 4, 6, 7 and 8 showed no significant (p>0.05) differences between baselines and day 14th but there were significant (p <0.05) increases between baselines and day 21. There were significant (p < 0.05) increases in Group 5 and 9 between baseline, day 14th and day 21”. In comparison between day 14th and day 21, only Group 4 and 8 increased significantly (p < 0.05) in HDL levels.

#### *Theobroma cacao* extracts lowered LDL levels in L-NAME induced hypertension

Induction of hypertension (Group 2) caused a significant (p <0.05) increase in LDL concentrations under hypertensive condition at days 14 and 21 when compared with group 1 (control) and baseline. However, concurrent treatments of L-NAME with ethanol extract (Groups 3, 4 and 5), chloroform extract (Groups 6,7and 8) and nifedipine (Group 9) produced significant (p <0.05) decreases in LDL levels compared with Group 2 (hypertensive untreated) and their baselines. Comparing between 14th and 21st days, Group 2 showed significant (p < 0.05) increase in LDL levels, Groups 4, 8 and 9 showed significant (p <0.05) decreases in LDL levels, Groups 3, 5, 6 and 7 showed non-significant (p > 0.05) differences but there were slight decreases in HDL levels.

#### *Theobroma cacao* extracts lowered very low-density lipoprotein concentrations in L-NAME induced hypertension

As shown in Table 4, induction of hypertension (Group 2) gave a significant (p < 0.05) increase in VLDL concentrations at the 14th and 21st days compared with Group 1 (control) and baseline. However, concurrent treatments of L-NAME with the ethanol extract (Groups 3, 4 and 5), chloroform extract (Groups 6, 7 and 8) and nifedipine (Group 9) showed significant (p < 0.05) decreases at 14th and 21st days relative to Group 2 (hypertensive untreated) and their baselines. Group 9 showed a slight decrease but not statistically significant in VLDL levels compared to the baseline. Comparing between 14th and 21 days, Group 2 showed non-significant (p>0.05) difference but there was a slight increase, there were decreases in all the treated groups but not statistically significant**.**

#### *Theobroma cacao* extracts lowered triacylglycerol levels in L-NAME induced hypertension

Induction of hypertension in Group 2 led to a significant (p <0.05) increase in triacylglycerol concentrations on days 14th and day 21st compared with Group 1 (control) and baseline. However, concurrent treatments of L-NAME with the ethanol extract (groups 3, 4 and 5), chloroform extract (Groups 6, 7 and 8) and nifedipine (Group 9) induced significant (p < 0.05) decreases in TAG levels compared with the Group 2 (hypertensive untreated) and their baselines. Comparing between 14th and 21st days, (Group 2 showed non-significant (p > 0.05) difference but there was a slight increase, there were decreases in all the treated groups but not statistically significant.

### Characterization of the ethanol and chloroform extracts of *T. cacao*

Characterization of the ethanol and chloroform extracts of *T. cacao* was carried out by HPLC to identify Quercetin (Retention time (RT): 11.050 min, 149.056), Kaempferol (RT:12.166 min, 48.103), chlorogenic acid (RT:1.266 min, 31.998) phenylacetic (RT:2.750 min, 23.544) and Theobromine (RT: 13.700 min, 15.781) as the top five compounds in the ethanol extract of *T. cacao* while Quercetin (RT:11.050 min, 138.754), (Kaempferol (RT:12.166 min, 38.494), Chlorogenic acid (RT:1.266 min, 21.889), Theobromine (RT:13.700 min, 17.508) and Gallic acid (RT:4.450 min, 12.909) as the five most abundant compounds in the chloroform extract of *T. cacao*).

## Discussion

The burden of cardiovascular disease (CVD) is increasing in Nigeria, driven by socio-economic factors such as rapid urbanization and lifestyle changes 26. CVDs now account for approximately 10% of all deaths and 3.8% of disability-adjusted life years (DALYs) in the country 27. Ischemic heart disease and stroke, with mortality rates of 105 and 91 deaths per 100,000 population respectively, rank as the second and fifth leading causes of age-specific mortality. From a current and future health perspective, hypertension is one of the most common and concerning forms of CVD. Its prevalence in Nigeria, estimated at 30.6% 28, represents a significant public health challenge requiring urgent attention. Given the alarming statistics, it is imperative for local researchers to intensify efforts in the search for therapeutics that are not only effective but also have minimal side effects, are readily available, and remain affordable. Plant-based therapeutics often meet these criteria and offer a promising alternative to conventional drugs. Notably, Nigeria and Africa at large is endowed with rich plant biodiversity, a resource largely attributed to its favorable climatic conditions. This abundance presents a valuable opportunity for the discovery and development of novel, plant-derived treatments for cardiovascular and other chronic diseases. In the present study, we investigated the potential of ethanol and chloroform extracts of *Theobroma cacao* seeds—a widely consumed seed known for its use in cocoa beverages and chocolate production—in attenuating oxidative stress and dyslipidemia in hypertensive albino rats.

The ethanol extract of *Theobroma cacao* (EETC) showed markedly stronger radical scavenging than the chloroform extract (CETC), with lower IC₅₀ values for both DPPH (59.9 µg/mL vs. 181.8 µg/mL) and ABTS (51.1 µg/mL vs. negligible inhibition, IC₅₀ not determinable), comparable to the standard BHT. In contrast, CETC displayed slightly better H₂O₂ scavenging (157.8 µg/mL vs. 182.1 µg/mL for EETC), though both were less potent than BHT (97.3 µg/mL). These patterns suggest that polar polyphenols in EETC are the main contributors to electron/hydrogen-donating activity, while some non-polar constituents contribute to peroxide quenching.

Both extracts also reduced nitrite accumulation in the Griess assay, confirming nitric oxide (NO•) scavenging in a concentration-dependent manner, with EETC being most effective. While NO is essential for vascular signaling, excessive production or ROS-mediated depletion contributes to endothelial dysfunction. By lowering radical burden, *T. cacao* extracts may preserve NO bioavailability and redox balance. Overall, these results highlight the dominant antioxidant capacity of the ethanol extract, providing a mechanistic basis for the in-vivo improvements in oxidative stress, lipid profile, and blood pressure observed in L-NAME-treated rats.

The pathogenesis of hypertension is complex and multifactorial, involving a wide range of physiological and environmental risk factors. Increasing evidence indicates that activation of the inflammatory response and the immune system plays a crucial role in the initiation and progression of hypertension. These immune-mediated mechanisms are also strongly associated with hypertensive complications, including myocardial infarction, hemorrhagic stroke, and renal injury 31. Therefore, the significance of anti-inflammatory agents in the management of hypertension can not be overemphasized. To evaluate the anti-inflammatory potential of EETC and CETC. three assays were conducted: inhibition of protein denaturation, Heat-induced hemolysis (HIH), and proteinase inhibition. Diclofenac sodium was used as the standard anti-inflammatory drug. In the protein denaturation assay, both extracts inhibited heat-induced albumin denaturation in a dose-dependent manner. The ethanol extract displayed stronger inhibition than the chloroform extract, achieving approximately 75% inhibition at 1000 μg/mL. Diclofenac exhibited the highest activity, exceeding 85% inhibition. These findings suggest that compounds in the ethanol extract may confer structural stability to proteins under stress conditions. The HIH assay revealed that the ethanol extract offered greater protection than the chloroform extract. At higher doses, the effect of the ethanol extract nearly approached that of diclofenac. The chloroform extract showed moderate inhibition, achieving roughly 50% protection at the maximum concentration. In the proteinase inhibition assay, the ethanol extract inhibited proteolytic activity more effectively than the chloroform extract, particularly at concentrations above 125 μg/mL. The ethanol extract achieved around 70% inhibition at the highest dose, while the chloroform extract peaked at about 50%. Diclofenac maintained consistently high inhibition across all concentrations tested.

Rats administered L-NAME exhibited significantly elevated total cholesterol, LDL, TAG, and VLDL levels, alongside reduced HDL, compared to baseline and control groups. Co-treatment with *T. cacao* extract and L-NAME significantly reduced total cholesterol, LDL, TAG, and VLDL levels while elevating HDL, in contrast to L-NAME-only rats. These findings suggest that *T. cacao* extract may contain hypocholesterolemic agents, consistent with previous reports that cocoa polyphenols reduce plasma cholesterol, increase HDL, and decrease LDL oxidation. Elevated cholesterol, TAG, and LDL levels, along with low HDL, are established risk factors for atherosclerosis and cardiovascular disease 32,33. The observed increase in TAG in hypertensive rats aligns with prior studies linking high TAG levels to elevated blood pressure 34,35. Furthermore, reductions in LDL and increases in HDL have been associated with regression of atherosclerotic lesions 36. Thus, *T. cacao* may offer cardiovascular protection in hypertensive conditions.

*T. cacao* extract significantly reduced malondialdehyde (MDA) levels in L-NAME-treated rats, indicating inhibition of lipid peroxidation. MDA, a mutagenic byproduct of oxidative lipid damage, is elevated in hypertension and contributes to vascular injury. Rats receiving *T. cacao* (100-300 mg/kg) or standard drugs alongside L-NAME showed a dose-dependent reduction in MDA and a significant increase in antioxidant enzymes (SOD, CAT, and GPx), compared to L-NAME-only and baseline groups. It was also observed that the effects of the extracts are not significantly different when compared to that of the standard drug. The observed oxidative stress in L-NAME-treated rats, marked by elevated MDA and suppressed antioxidant activity, aligns with previous reports linking ROS overproduction to hypertension pathogenesis 37,38. *T. cacao*'s antioxidant effects may be mediated by upregulating endogenous defense enzymes via Nrf2/ARE signaling, counteracting NADPH oxidase-driven ROS generation 39,40 By restoring redox balance, *T. cacao* demonstrated greater protection than the standard drug, suggesting its therapeutic potential in preventing oxidative stress-related vascular damage in hypertension.

### Limitations

Although we did not include extract‑only arms in normotensive rats, prior studies have reported effects of *Theobroma cacao* in normotensive models; nonetheless, we will include normotensive extract‑only cohorts in follow‑up experiments to quantify resting SBP/DBP/MAP, heart rate, lipid indices, and redox markers independent of L‑NAME. In addition, only male rats were studied; future work will enroll matched female cohorts and prespecified sex‑stratified analyses to assess potential sex differences in antioxidant and antihypertensive responses.

## Conclusion

In conclusion, ethanol and chloroform extracts of *Theobroma cacao* mitigate L-NAME-induced hypertension by restoring antioxidant defenses, reducing lipid peroxidation, and improving serum lipid profiles. These results support the therapeutic potential of *T. cacao* seed extracts for oxidative stress-related cardiovascular conditions. Future studies should focus on identifying the active constituents, determining optimal doses, and assessing their clinical effectiveness.

## Figures and Tables

**Figure 4:**
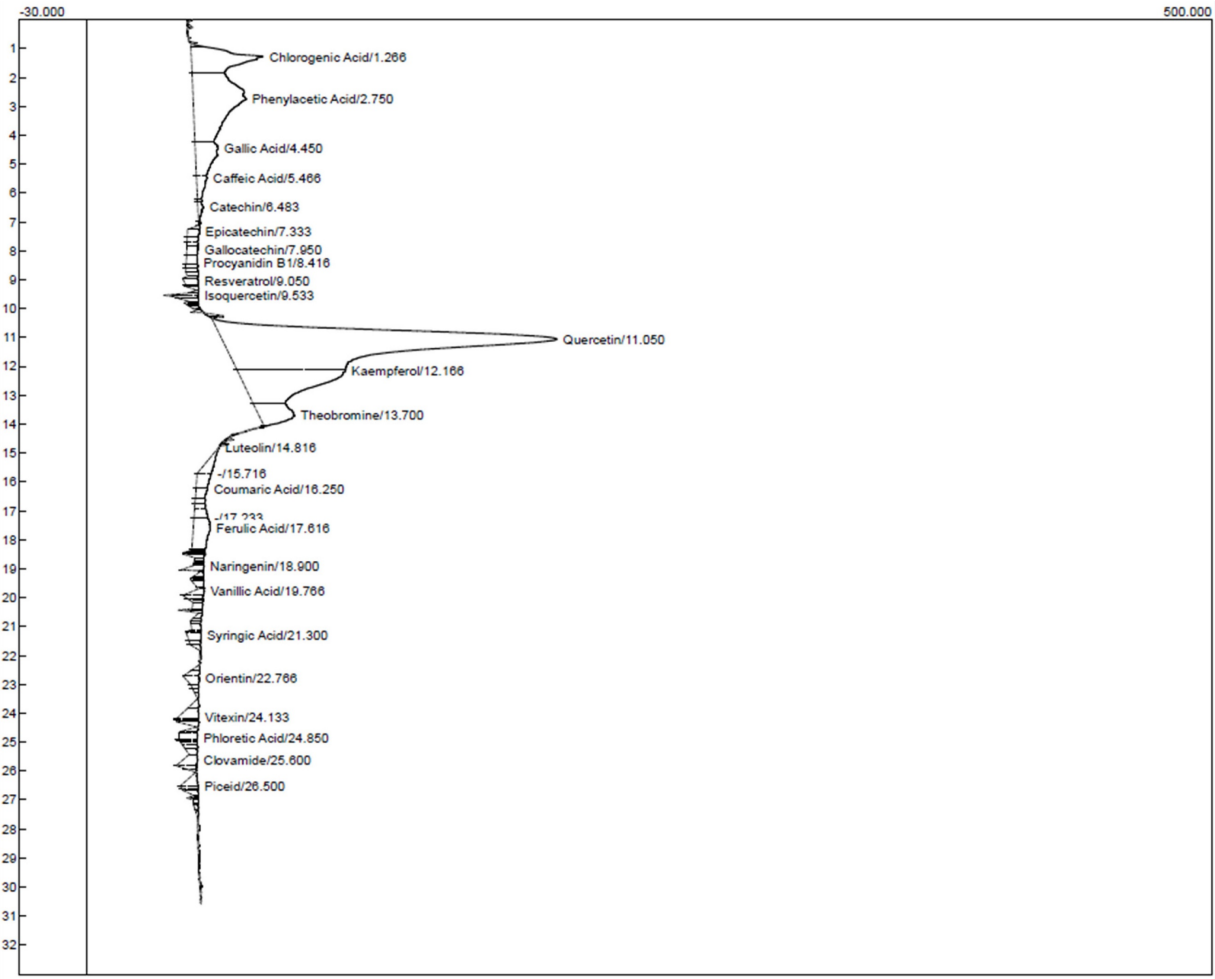
HPLC chromatogram for the ethanol extract of *T. cacao*.

**Figure 5:**
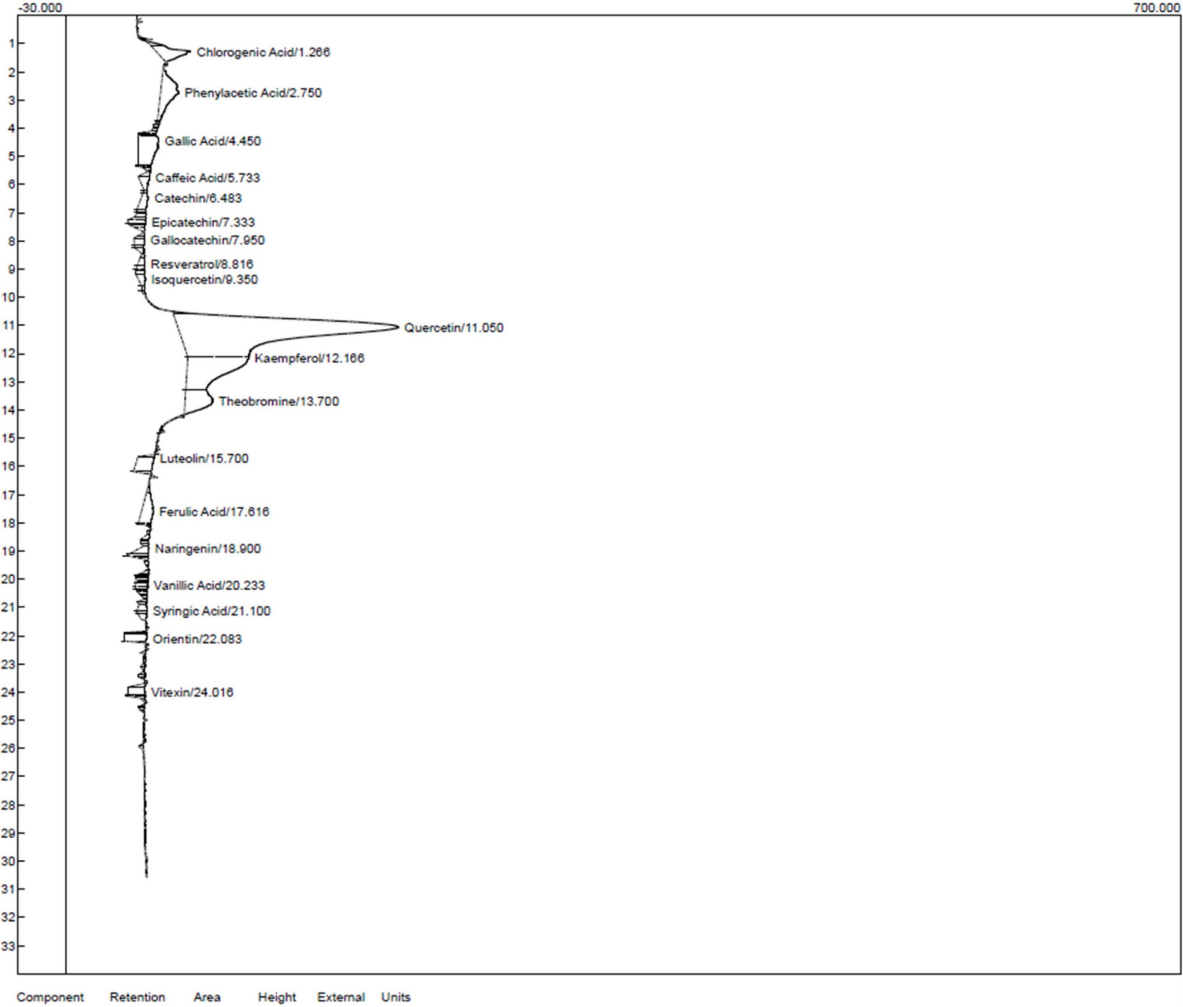
HPLC chromatogram for the chloroform extract of *T. cacao*.

**Table 1:** IC_50_ values for *in vitro* antioxidants and anti-inflammatory assays of EETC and CETC

Assay	Chloroform	Ethanol	Standard Drug(s)
**ABTS Assay**	0.00 ± 0.00*	51.05 ± 2.87	BHT: 9.39 ± 0.21
**DPPH Assay**	181.79 ± 22.90	59.92 ± 0.95	BHT: 71.49 ± 2.42
**H₂O₂ Scavenging**	157.76 ± 12.66	182.11 ± 5.67	BHT: 97.25 ± 1.78
**Proteinase Inhibition**	190.73 ± 18.16	172.81 ± 15.73	Diclofenac: 74.52 ± 8.13
**Protein Denaturation**	105.76 ± 2.18	181.37 ± 10.88	Diclofenac: 21.84 ± 0.42
**HIH (Membrane Stability)**	381.65 ± 15.50	169.45 ± 8.32	Diclofenac: 83.13 ± 2.62

**Table 2:** Effect of the extracts on MDA concentrations in L-NAME-induced hypertensive rats

Treatment Group	MDA (U/mgProtein)		
	Baseline	Day 14	Day 21
Group 1	2.9±0.1	2.0±0.1	1.4±0.3
Group 2	3.4±0.3	5.0±0.6^a^	5.8±0.6^af^
Group 3	3.1±0.3	2.0±0.1^bd^	1.9±0.1^bcd^
Group 4	3.3±0.4	2.1±0.1^bd^	0.80±0.1^bcd^
Group 5	3.2±0.4	2.1± 0.2^bd^	0.6±0.0^bcde^
Group 6	2.2±0.0	1.2±0.8^bde^	1.1±0.2^bd^
Group 7	2.4±0.1	1.2±0.1^bde^	0.9±0.1^bd^
Group 8	2.8±0.2	1.5±0.2^bde^	0.4±0.1^bcd^
Group 9	3.3±0.5	2.0±0.1^bd^	1.0±0.2^bcd^

**Table 3:** Effect of the extracts on GPx activities in L-NAME-induced hypertensive rats

Treatment Group	Glutathione Peroxidase Activities (U/mgProtein)		
	Baseline	Day 14	Day 21
Group 1	27.4±0.2	21.3±2.9	23.1±0.9
Group 2	29.7±1.5	24.1±2.6	11.8±0.4^af^
Group 3	22.2±0.9	37.4±3.5^bd^	50.1±1.8^bcd^
Group 4	28.0±5.2	40.3±3.4^b^	53.6±6.8^bcd^
Group 5	39.1±1.9	49.9±1.0^bde^	60.1±3.0^bcde^
Group 6	35.1±5.51	42.7±3.5^b^	46.9±5.8^bd^
Group 7	24.54±5.03	36.5±1.7^bd^	49.7±3.8^bcd^
Group 8	24.9±4.5	42.5±1.0^bd^	51.0±0.9^bcd^
Group 9	23.6±3.8	38.0±3.6^bd^	48.0±0.5^bcd^

Results are means ± SEM (n = 3). Means values with different letters as superscripts across the row and columns are considered significant at p < 0.05

**Table 4:** Effect of the extracts on SOD activities in L-NAME- induced hypertensive rats

Treatment Group	SOD (U/mgProtein)		
	Baseline	Day 14	Day 21
Group 1	67.6±19.3	64.9±12.2	64.4±42.5
Group 2	84.3±4.8	54.4±12.5a	23.8±2.7af
Group 3	58.5±12.6	74.0±14.1^d^	85.3±45.8^bd^
Group 4	64.9±31.0	83.0±3.4^b^	94.1±31.5^b^
Group 5	76.5±25.1	84.68±2.4^bd^	150.6±9.9^bcde^
Group 6	89.2±9.9	86.5±16.6^b^	88.6±19.6^b^
Group 7	78.1±26.6	84.7 ± 2.0^b^	116.1±10.3^b^
Group 8	88.6±7.3	90.3±18.0^b^	114.1±6.6^b^
Group 9	89.9±24.9	89.5±4.4^b^	99.7±10.7^b^

Results are means ± SEM (n = 3). Means values with different letters as superscripts across the row and columns are considered significant at p < 0.05

**Table 5:** Effect of the extracts on CAT activities in L-NAME-induced hypertensive rats

Treatment Group	CAT (U/mgProtein)		
	Baseline	Day 14	Day 21
Group 1	51.6±0.6	44.8±3.0	64.7±12.3
Group 2	68.3±4.8	38.1±5.6^a^	24.3±1.9^af^
Group 3	50.1±2.3	56.4±4.0^b^	63.5±2.8^bd^
Group 4	50.5±4.9	60.1±2.5^bd^	67.9±5.1^bd^
Group 5	53.9±1.9	63.5±1.9^bd^	71.0±4.7^bcde^
Group 6	42.6±3.8	60.4±10.1^bd^	63.0±9.2^bd^
Group 7	48.8±6.0	58.9±3.0^b^	66.6±6.3^bde^
Group 8	54.7± 6.4	58.4±4.3^b^	55.1±1.9^b^
Group 9	53.1±8.3	57.1±4.2^b^	54.3±8.6^b^

Results are means ± SEM (n = 3). Means values with different letters as superscripts across the row and columns are considered significant at p < 0.05

**Table 6:** Effect of the extracts on nitric oxide concentrations in L-NAME- induced hypertensive rats

Treatment Group	Nitric Oxide (%)		
	Baseline	Day 14	Day 21
Group 1	44.0±0.3	32.6±5.5	36.5±0.4
Group 2	41.1± 3.1	21.4±12.3^a^	14.8±3.7^a^
Group 3	41.0±2.3	29.0±4.3	59.9±1.0^bcd^
Group 4	35.3±12.4	42.6±4.4^bc^	60.8±4.8^bcd^
Group 5	29.9±8.6	45.5±2.9^bcd^	69.4±2.7^bcde^
Group 6	36.7±0.9	42.2±2.4^bc^	64.1±2.0^bcd^
Group 7	41.5±3.2	45.5±1.3^bc^	64.6±1.1^bcd^
Group 8	56.3±0.2	58.9±4.7^bc^	66.2±1.7^bc^
Group 9	48.9±0.2	54.2±1.3^bd^	61.7±1.8^bcd^

Results are means ± SEM (n = 3). Means values with different letters as superscripts across the row and columns are considered significant at p < 0.05

**Table 7:** Effect of the extracts on cholesterol concentration in L-NAME-induced hypertensive rats

Treatment Group	Total Cholesterol Concentrations (mg∕dl)		
	Baseline	Day 14	Day 21
Group 1	74.5±15.7	79.46±25.90	71.60±1.43
Group 2	116.0±6.0	122.66±7.05	156.3±3.8^ac^
Group 3	118.1±5.2	104.8±0.0^bd^	85.6±8.4^bd^
Group 4	116.3±0.0	91.73±6.99^bde^	68.3±4.4^bde^
Group 5	116.3±20.9	81.06±4.36^bde^	53.5±1.0^bde^
Group 6	113.3±15.1	95.8±4.36^ bde^	82.3±5.2^bde^
Group 7	96.9±8.9	77.3±7.3^bde^	63.9±8.2^bde^
Group 8	116.3±5.2	77.3±6.4^ bde^	54.1±2.8^bde^
Group 9	116.3±5.2	105.7±1.9^b^	82.1±1.6^bcd^

Results are means ± SEM (n = 3). Means values with different letters as superscripts across the row and columns are considered significant at p < 0.05

**Table 8:** Effect of the extracts on HDL concentration in L-NAME- induced hypertensive rats

Treatment Group	HDL Conc (mg/dl)		
	Baseline	Day 14	Day 21
Group 1	49.0±1.8	47.7±11.0	47.2±3.3
Group 2	48.1±4.5	29.5±0.9^a^	21.7±2.5^af^
Group 3	41.7±7.4	51.5±4.3^bde^	56.4±7.0^bde^
Group 4	47.2±6.3	54.3±3.3^bd^	62.8±8.8^bcd^
Group 5	47.2±8.6	63.8±4.1^bd^	76.6±7.9^bcde^
Group 6	43.5±4.1	53.4±2.5^bd^	66.4±11.3^bd^
Group 7	37.2±3.9	54.3±5.7^bd^	70.2±1.3^bd^
Group 8	49.9±7.2	55.3±13.9^bd^	73.30±8.3^bcd^
Group 9	48.1±2.3	62.9±2.8^bd^	66.4±4.5^bd^

Results are means ± SEM (n = 3). Means values with different letters as superscripts across the row and columns are considered significant at p < 0.05

**Table 9:** Effect of the extracts on LDL concentrations in L-NAME-induced hypertensive rats

Treatment Group	LDL Concentrations (mg/dl)		
	Baseline	Day 14	Day 21
Group 1	22.6±1.8	22.9±12.8	7.6±4.2
Group 2	39.7±1.8	65.1±5.5^a^	103.2±8.9^af^
Group 3	54.2±9.4	17.9±3.8^bde^	14.6±2.1^bd^
Group 4	78.9±6.1	24.5±4.5^bde^	8.6±2.8^bd^
Group 5	70.4±21.6	5.3±0.9^bde^	3.3±0.6^bde^
Group 6	67.6±20.9	18.2±5.8^bde^	7.9±2.5^bd^
Group 7	49.9±23.5	13.9±4.2^bde^	9.4±3.0^bd^
Group 8	69.8±11.9	22.3±6.0^bde^	4.5±0.8^bcd^
Group 9	61.0±6.1	38.8±0.3^bd^	8.7±1.4^bcd^

Results are means ± SEM (n = 3). Means values with different letters as superscripts across the row and columns are considered significant at p < 0.05

**Table 10:** Effect of the extracts on VLDL concentration in L-NAME- induced hypertensive rats

Treatment Group	VLDL Concentrations (mg/dl)		
	Baseline	Day 14	Day 21
Group 1	45.0±2.2	34.6±3.6	37.3±11.9
Group 2	40.6±1.1	62.4±8.0^a^	70.0±11.0^af^
Group 3	49.4±3.2	34.3±2.3^bd^	23.6±3.8^bd^
Group 4	45.0±1.1	28.5±2.7^bd^	19.8±3.8^bde^
Group 5	41.7±2.2	26.4±1.7^bd^	19.0±0.7^abe^
Group 6	49.4±6.5	31.7±3.2^bd^	23.6±4.1^bd^
Group 7	51.6±2.9	32.0±3.3^bd^	22.8±2.8^bd^
Group 8	59.3±1.9	29.1±2.7^bd^	19.8±1.1^bcde^
Group 9	38.4±1.1	31.2±2.7^b^	27.5±1.9^bcd^

Results are means ± SEM (n = 3). Means values with different letters as superscripts across the row and columns are considered significant at p < 0.05

**Table 11:** Effect of the extracts on TAG concentration in L-NAME-induced hypertensive rats

Treatment Group	Triacylglyceride Concentrations (mg/dl)		
	Baseline	Day 14	Day 21
Group 1	100.9±4.9	77.5±8.09	83.5±26.8
Group 2	91.0 ±2.5	139.7±17.9^a^	156.7±24.8^a^
Group 3	110.8±7.4	76.9±5.2^bd^	53.0±8.6^bd^
Group 4	100.9±2.5	64.0±6.1^bd^	44.5±8.5^bde^
Group 5	93.5±4.9	59.3±3.8^bd^	42.6±1.6^bcde^
Group 6	110.8±14.8	71.0±7.3^bd^	53.0±9.1^bd^
Group 7	115.7±6.5	71.6±7.4^bd^	51.2±6.3^bd^
Group 8	132.9±4.3	65.1±6.0^bd^	44.5±2.6^bd^
Group 9	86.13±2.5	96.8±6.2^b^	61.5±4.4^b^

Results are means ± SEM (n = 3). Means values with different letters as superscripts across the row and columns are considered significant at p < 0.05

**Table 12:** HPLC characteristics of the compounds identified in the ethanol extract of *T. cacao*

Components	Retention	Area	Height
Chlorogenic acid	1.266	1060.7840	31.998
Phenylacetic acid	2.750	2310.5790	23.544
Gallic acid	4.450	552.6750	10.070
Caffeic acid	5.466	156.8410	4.769
Catechin	6.483	61.0475	2.545
Epicatechin	7.333	81.9950	5.385
Gallocatechin	7.950	102.3055	5.087
Procyanidin B1	8.416	82.3060	5.048
Resveratrol	9.050	91.7960	6.513
Isoquercetin	9.533	60.2155	15.439
Quercetin	11.050	80005.2720	149.056
Kaempferol	12.166	2203.0820	48.103
Theobromine	13.700	582.7050	15.781
Luteolin	14.816	175.3440	0.439
Coumaric acid	16.250	103.2780	5.271
Ferulic acid	17.616	435.2880	7.511
Naringenin	18.900	65.2410	5.403
Vanillic acid	19.766	77.7860	4.859
Syringic acid	21.300	81.9340	6.247
Orientin	22.766	95.6350	6.247
Vitexin	24.133	167.6375	9.239
Phloretic acid	24.850	110.1630	8.575
Clovamide	25.600	148.0165	6.352
Piceid	26.500	113.6550	7.659

**Table 13:** HPLC characteristics of the compounds identified in the chloroform extract of *T. cacao*

Components	Retention	Area	Height
Chlorogenic acid	1.266	401.1120	21.889
Phenylacetic acid	2.750	649.3350	11.407
Caffeic acid	4.450	695.4735	12.909
Catechin	5.733	134.3900	6.968
Epicatechin	7.333	75.7995	10.854
Gallocatechin	7.950	100.7250	6.704
Resveratrol	8.816	63.1560	4.461
Isoquercetin	9.350	87.7320	4.422
Quercetin	11.050	6961.9475	138.754
Kaempferol	12.166	1805.1270	38.494
Theobromine	13.700	671.3320	17.508
Luteolin	15.700	342.5560	10.504
Ferulic acid	17.616	389.7360	7.413
Naringenin	18.900	117.8750	5.483
Vanillic acid	20.233	70.0745	7.886
Syringic acid	21.100	81.9530	6.214
Orientin	22.083	196.9240	14.303
Vitexin	24.016	166.0760	10.662

## References

[1] Mills KT, Stefanescu A, He J (2020). The global epidemiology of hypertension. Nat Rev Nephrol.

[2] Mowry FE, Biancardi VC (2019). Neuroinflammation in hypertension: the renin-angiotensin system versus pro-resolution pathways. Pharmacol Res.

[3] Stanaway JD, Afshin A, Gakidou E, Lim SS, Abate D, Abate KH (2018). Global, regional, and national comparative risk assessment of 84 behavioural, environmental and occupational, and metabolic risks or clusters of risks for 195 countries and territories, 1990-2017: a systematic analysis for the Global Burden of Disease Study 2017. The Lancet.

[4] Carey RM, Muntner P, Bosworth HB, Whelton PK (2018). Prevention and Control of Hypertension. J Am Coll Cardiol.

[5] Berberich AJ, Hegele RA (2022). A Modern Approach to Dyslipidemia. Endocr Rev.

[6] Ballard-Hernandez J, Sall J (2023). Dyslipidemia Update. Nursing Clinics of North America.

[7] S.K (2023). Masenga, L.S. Kabwe MCAK, M. Chakulya, M. Chakulya AK. Mechanisms of oxidative stress in metabolic syndrome. Int J Mol Sci.

[8] Koju N, Taleb A, Zhou J, Lv G, Yang J, Cao X (2019). Pharmacological strategies to lower crosstalk between nicotinamide adenine dinucleotide phosphate (NADPH) oxidase and mitochondria. Biomedicine and Pharmacotherapy.

[9] Vekic J, Stromsnes K, Mazzalai S, Zeljkovic A, Rizzo M, Gambini J (2023). Oxidative Stress, Atherogenic Dyslipidemia, and Cardiovascular Risk. Biomedicines.

[10] Shahidi F, Zhong Y (2011). Revisiting the Polar Paradox Theory: A Critical Overview. J Agric Food Chem [Internet].

[11] Prasathkumar M, Anisha S, Dhrisya C, Becky R, Sadhasivam S (2025). Therapeutic and pharmacological efficacy of selective Indian medicinal plants - A review. Phytomedicine Plus [Internet]. 2021 May 1 [cited.

[12] Soares T F, Oliveira M B P P (2022). Cocoa by-products: characterization of bioactive compounds and beneficial health effects. Molecules.

[13] Tonisi S, Okaiyeto K, Hoppe H, Mabinya L V, Nwodo UU, Okoh AI (2020). Chemical constituents, antioxidant and cytotoxicity properties of Leonotis leonurus used in the folklore management of neurological disorders in the Eastern Cape, South Africa. 3 Biotech.

[14] BLOIS MS (1958). Antioxidant Determinations by the Use of a Stable Free Radical. Nature.

[15] Ruch RJ, Cheng SJ, Klaunig JE (1989). Prevention of cytotoxicity and inhibition of intercellular communication by antioxidant catechins isolated from Chinese green tea. Carcinogenesis.

[16] Juvekar A, Sakat S, Wankhede S, Juvekar M, Gambhire M (2009). Evaluation of antioxidant and anti-inflammatory activity of methanol extract of Oxalis corniculata. Planta Med.

[17] Oyedapo OO, Famurewa AJ (1995). Antiprotease and Membrane Stabilizing Activities of Extracts of Fagara Zanthoxyloides, Olax Subscorpioides and Tetrapleura Tetraptera. International Journal of Pharmacognosy.

[18] Mizushima Y, Kobayashi M (1968). Interaction of anti-inflammatory drugs with serum proteins, especially with some biologically active proteins. Journal of Pharmacy and Pharmacology.

[19] Mainasara AS, Isa SA, Dandare A, Ladan MJ, Saidu Y, Rabiu S (2016). Blood pressure profile and insulin resistance in salt-induced hypertensive rats treated with camel milk. Med J Nutrition Metab.

[20] Friedewald WT, Levy RI, Fredrickson DS (1972). Estimation of the concentration of low-density lipoprotein cholesterol in plasma, without use of the preparative ultracentrifuge. Clin Chem.

[21] Albers JJ, Warnick GR, Chenng MC (1978). Quantitation of high density lipoproteins. Lipids.

[22] Bergmeyer HU (1974). Methods of Enzymatic Analysis. 2nd ed. New York.: Academic Press.

[23] Misra HP, Fridovich I (1972). The role of superoxide anion in the autoxidation of epinephrine and a simple assay for superoxide dismutase. J Biol Chem.

[24] Wallin B, Rosengren B, Shertzer HG, Camejo G (1993). Lipoprotein Oxidation and Measurement of Thiobarbituric Acid Reacting Substances Formation in a Single Microtiter Plate: Its Use for Evaluation of Antioxidants. Anal Biochem.

[25] Marcocci L, Maguire JJ, Droylefaix MT, Packer L (1994). The Nitric Oxide-Scavenging Properties of Ginkgo Biloba Extract EGb 761. Biochem Biophys Res Commun.

[26] Ogah OS, Orimolade OA, Jinadu TO (2023). Cardiovascular Diseases in Nigeria: Current Status, Threats, and Opportunities. Circulation.

[27] Murray CJL, Aravkin AY, Zheng P, Abbafati C, Abbas KM, Abbasi-Kangevari M (2020). Global burden of 87 risk factors in 204 countries and territories, 1990–2019: a systematic analysis for the Global Burden of Disease Study 2019. The Lancet [Internet].

[28] Ogah OS, Stewart S, Falase AO, Akinyemi JO, Adegbite GD, Alabi AA (2014). Contemporary Profile of Acute Heart Failure in Southern Nigeria. JACC Heart Fail.

[29] Chaudhary P, Janmeda P, Docea AO, Yeskaliyeva B, Abdull Razis AF, Modu B (2023). Oxidative stress, free radicals and antioxidants: potential crosstalk in the pathophysiology of human diseases. Front Chem.

[30] Yeo SG, Oh YJ, Lee JM, Kim SS, Park DC (2025). A Narrative Review of the Expression and Role of Nitric Oxide in Endometriosis. Antioxidants.

[31] Barrows IR, Ramezani A, Raj DS (2019). Inflammation, Immunity, and Oxidative Stress in Hypertension—Partners in Crime?. Adv Chronic Kidney Dis.

[32] Linton MF, Yancey PG, Davies SS, Jerome WG, Linton EF, Song WL Endotext [Internet]. 2019. The role of lipids and lipoproteins in atherosclerosis.

[33] Borén J, Chapman MJ, Krauss RM, Packard CJ, Bentzon JF, Binder CJ (2020). Low-density lipoproteins cause atherosclerotic cardiovascular disease: pathophysiological, genetic, and therapeutic insights: a consensus statement from the European Atherosclerosis Society Consensus Panel. Eur Heart J.

[34] Shimizu Y, Sato S, Noguchi Y, Koyamatsu J, Yamanashi H, Nagayoshi M (2017). Triglycerides and blood pressure in relation to circulating CD34-positive cell levels among community-dwelling elderly Japanese men: a cross-sectional study. Environ Health Prev Med.

[35] Li B, He X, Lei SS, Zhou FC, Zhang NY, Chen YH (2019). Hypertensive Rats Treated Chronically With Nω-Nitro-L-Arginine Methyl Ester (L-NAME) Induced Disorder of Hepatic Fatty Acid Metabolism and Intestinal Pathophysiology. Front Pharmacol.

[36] Ndrepepa G (2021). High-density lipoprotein: a double-edged sword in cardiovascular physiology and pathophysiology. J Lab Precis Med.

[37] Tain YL, Hsu CN (2022). Oxidative Stress-Induced Hypertension of Developmental Origins: Preventive Aspects of Antioxidant Therapy. Antioxidants.

[38] CHIA T, MURUGAIYAH V, KHAN N, SATTAR M, ABDULLA M, JOHNS E (2021). Inhibition of L-NAME-Induced Hypertension by Combined Treatment With Apocynin and Catalase: The Role of Nox 4 Expression. Physiol Res.

[39] Ngo V, Duennwald ML (2022). Nrf2 and Oxidative Stress: A General Overview of Mechanisms and Implications in Human Disease. Antioxidants.

[40] Jomova K, Alomar SY, Alwasel SH, Nepovimova E, Kuca K, Valko M (2024). Several lines of antioxidant defense against oxidative stress: antioxidant enzymes, nanomaterials with multiple enzyme-mimicking activities, and low-molecular-weight antioxidants. Arch Toxicol.

